# Data Processing and Quality Evaluation of a Boat-Based Mobile Laser Scanning System

**DOI:** 10.3390/s130912497

**Published:** 2013-09-17

**Authors:** Matti Vaaja, Antero Kukko, Harri Kaartinen, Matti Kurkela, Elina Kasvi, Claude Flener, Hannu Hyyppä, Juha Hyyppä, Juha Järvelä, Petteri Alho

**Affiliations:** 1 Department of Real Estate, Planning and Geoinformatics, Aalto University, Aalto FI-00076, Finland; E-Mails: Matti.Kurkela@aalto.fi (M.K.); Hannu.Hyyppa@aalto.fi (H.H.); mipeal@utu.fi (P.A.); 2 Department of Remote Sensing and Photogrammetry, Finnish Geodetic Institute, P.O. Box 15, Masala FI-02431, Finland; E-Mails: Antero.Kukko@fgi.fi (A.K.); Harri.Kaartinen@fgi.fi (H.K.); Juha.Hyyppä@fgi.fi (J.H.); 3 Department of Geography and Geology, University of Turku, Turku FI-20014, Finland; E-Mails: emkasv@utu.fi (E.K.); Claude.Flener@utu.fi (C.F.); 4 Civil Engineering and Building Services, Helsinki Metropolia University of Applied Sciences, Metropolia FI-00079, Finland; 5 Department of Civil and Environmental Engineering, Aalto University, Aalto FI-00076, Finland; E-Mail: Juha.Jarvela@aalto.fi

**Keywords:** mobile laser scanning, ground point classification, data processing, digital elevation model, filtering, river survey, fluvial geomorphology

## Abstract

Mobile mapping systems (MMSs) are used for mapping topographic and urban features which are difficult and time consuming to measure with other instruments. The benefits of MMSs include efficient data collection and versatile usability. This paper investigates the data processing steps and quality of a boat-based mobile mapping system (BoMMS) data for generating terrain and vegetation points in a river environment. Our aim in data processing was to filter noise points, detect shorelines as well as points below water surface and conduct ground point classification. Previous studies of BoMMS have investigated elevation accuracies and usability in detection of fluvial erosion and deposition areas. The new findings concerning BoMMS data are that the improved data processing approach allows for identification of multipath reflections and shoreline delineation. We demonstrate the possibility to measure bathymetry data in shallow (0–1 m) and clear water. Furthermore, we evaluate for the first time the accuracy of the BoMMS ground points classification compared to manually classified data. We also demonstrate the spatial variations of the ground point density and assess elevation and vertical accuracies of the BoMMS data.

## 1. Introduction

Laser scanning (LS) is widely used to create accurate 3-D descriptions such as digital elevation models (DEMs) for natural and urban environments. LS methods can be divided into three main categories: airborne laser scanning (ALS), terrestrial laser scanning (TLS) and mobile laser scanning (MLS). The latter has become a common technique when objects need to be modelled from a terrestrial point of view. Data provided by an MLS system can be characterized by a point density in the range of 100–1,000 pulses per m^2^ at a 10 m distance from the scanner and with an operational scanning range between 1 and 100 m [1]. With the use of improved georeferencing and calibration of systems, mobile laser scanning in controlled conditions can achieve elevation and planimetric accuracies (std) of 1–4 cm [2]. An overview of mobile mapping systems is presented by El-Sheimy [3], Graham [4] and Petrie [5].

In order to reliably determine the terrain information for the DEM creation, filtering of the LS data, *i.e.*, classification of the point cloud into terrain and non-terrain points, is essential. Many algorithms have been developed for filtering ALS data, but these approaches are also relevant to the filtering of other point cloud data, e.g., TLS, multibeam echo sound data or point clouds obtained by automated image matching [6–8]. The filtering methods include morphological filters [9–12], densification methods [13,14], surface based filters [15,16] and segmentation based filters [17,18]. In the case of MLS data, higher point density has to be considered, for example when selecting the parameters of the algorithm. In many cases, the huge volume of MLS data requires it to be divided into blocks. In addition, trajectory information can be utilized for DEM generation. Leslar *et al.* [19] combined two different types of algorithms for the detection and filtering of outliers in point clouds collected using MLS. The practical performance of different algorithms is tested with an international filter test [20]. A good overview of the ground filtering methods used is presented by Meng *et al.* [21].

MLS products such as DEM or 3D-maps require effective and automatic processing methods. The studies of usability and data processing of MLS data have mainly focused on urban environments, *i.e.*, [22–25]. Performance of a mobile mapping system in urban areas has been evaluated by Haala *et al.* [26]. In natural landscapes the MLS-based applications have been discussed in the context of tree inventory [27,28], coastal mapping [29,30] and fluvial geomorphology [31]. The state-of-the-art multi-platform MLS systems and their usability and performance have been discussed by Kukko *et al.* [32].

Recently, the use of LS data in fluvial studies has rapidly increased. Detailed DEMs derived from LS data can be used to improve the recognition of fluvial landforms, the geometric data of hydraulic modelling, and the estimation of flood inundation extents and fluvial processes [33]. Furthermore, LS techniques have been considered promising for the hydraulic analyses of riverbank and floodplain vegetation [34,35]. However, there are only few MLS-based studies related to river environment. One of the newest applications for riverine mapping is the boat-based mobile mapping system (BoMMS). The BoMMS is a system primarily developed for urban mapping [36,37], but it has been frequently used for environmental applications [32,38,39]. The system enabled surveying a reach of approximately six kilometres in length in 85 min [38]. In our previous studies [31,38,39], we evaluated the use of BoMMS data for detecting fluvial erosion and deposition areas. The quality of BoMMS-produced DEMs and elevation difference maps compared to TLS control data has also been assessed. Despite previous studies, its full potential and data characteristics related to river environments should be studied in more detail.

The primary aim of this paper is to develop a data processing approach that is capable of generating terrain and vegetation points from BoMMS data while allowing for the identification of multipath reflections, delineation of shoreline, and consideration of bathymetry data. The secondary aim is to evaluate the accuracy of the BoMMS ground points classification compared to manually classified data. In addition, we demonstrate the spatial variations of the ground point density and assess elevation and horizontal accuracies of the BoMMS data.

## 2. Study Site and Data Acquisition

### 2.1. Test Site

The test site is located along the River Pulmankijoki, a 58 km-long tributary of the Tana River in the sub-Arctic, flowing across the border between Finland and Norway at 69.95° N latitude and 28.10° E longitude where Lake Pulmankijärvi divides the river into two parts. On the Finnish side, the river builds up a small delta into the lake. The river has eroded a 30-m deep and 20 to 50-m wide channel into glacio-fluvial sediments. The river is characterized by steep banks, sensitivity to erosion, sandbars and bushy vegetation. During the spring flood period caused by the snowmelt, the water level can be several meters higher than in autumn. The seasonal discharge ranges from 4 to 50 m^3^/s so that the typical spring flood discharge is ∼40–50 m^3^/s decreasing to 4–10 m^3^/s by the middle of June. The geomorphology of the study site is described in detail by Kasvi *et al.* [40] and Alho and Mäkinen [41].

### 2.2. Mobile Laser Data Collection with the BoMMS

**Figure 1.**
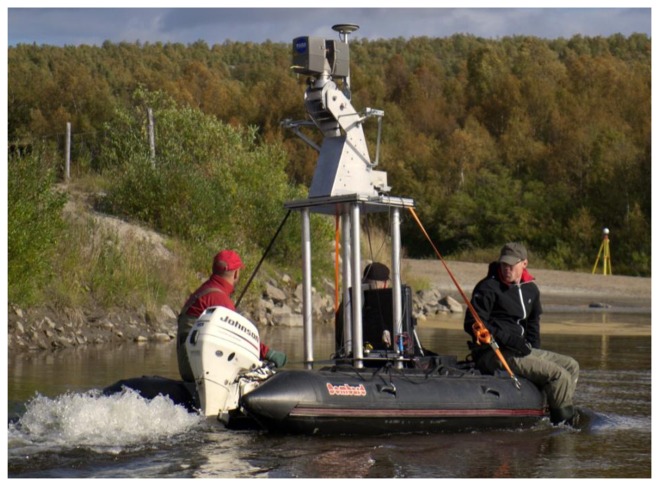
The boat-based mobile laser scanner used in the study.

The BoMMS measurements were conducted at the Pulmankijoki River site in late summer (late August–early September) in 2010, as during that time the water level was at its lowest and the non-vegetated point bars were as visible as possible. However, during this season, low vegetation is dense along the other part of the channel, reducing the number of laser pulses returning from bare ground. Figure 1 shows the BoMMS system on a boat installation and Table 1 presents the system parameters used in the data collection.

The nominal boat speed during data acquisition was 1–2 m/s, where the along-track point spacing (*d_along_*) can be calculated as:

(1) $$d_{\text{along}}=v/f_{sc}$$

where *v* is the platform speed and *f_sc_* is the scanning frequency [42]. The point spacing of adjacent points (*d_across_*) in a profile projected on a plane perpendicular to the beam at the range *r* is given by:

(2) $$d_{\text{across}}=2\pi rf_{sc}/f_{pr}$$

where *f_pr_* is the pulse repetition frequency. The typical mapping range of the BoMMS in this study varied from 3 m to 50 m.

The BoMMS consists of a global positioning system and inertial measurement unit (GPS-IMU) navigation system and a laser scanner combined with data synchronization and recording devices. Panasonic Toughbooks (CF-19 and CF-29) were used for scanner and navigation system operation and recording. Optionally, a camera system can be added for extraction of color information from the object. The GPS-IMU system uses the NovAtel DL-4plus receiver and a GPS-702 antenna capable of receiving L1 and L2 frequencies. The inertial measurement unit (IMU) employed is the Honeywell tactical-grade HG1700 AG58 IMU based on ring laser gyro (RLG). The GPS-IMU provides position and attitude data at 100 Hz for georeferencing the laser data in post-processing. The absolute error of the BoMMS is mainly dependent on the GPS-IMU navigation solution that can be provided in real-time, or more accurately through post-processing by means of the tactical-grade GPS-IMU. Another major impact on performance is a system calibration. Hence, the accuracy can be improved by performing a field calibration, *i.e.*, using test field TLS data in estimating the bore-sight parameters of the MLS system [32]. For GPS correction, GPS reference station data is typically downloaded from a virtual GPS network service, or a standalone reference station is used in places outside the virtual network stations. In Pulmanki reach, a standalone reference station was used. The distance of the baseline from the reference station to the system varied from 0.5 km to 1.5 km.

The BoMMS uses the FARO Photon 120 scanner providing a scan frequency range of 3–61 Hz and a point measurement rate of 120–976 kHz with maximum range of 150 m. Point measurement accuracy of the scanner is 2 mm with 1 mm repeatability for a 90% reflective target, according to the scanner manufacturer, but that depends on the object surface type, reflectivity and angle of incidence in practice.

### 2.3. Reference Data

To assess the BoMMS data quality of the z- and xy-coordinates, we installed spherical targets along the river reach. Two different target sizes were used, 145 mm and 198 mm in diameter. The spheres were positioned with RTK-GPS using the same reference station as BoMMS. RTK-GPS measurements enable a 10 mm + 1–2 ppm horizontal and 15–20 mm + 2 ppm elevation accuracy for the sphere target locations [43]. We measured the larger spheres by replacing the sphere with GPS-antenna on a common mount. The positions of the smaller spheres were measured from the top of the sphere, and we applied pre-defined calibration offsets between the centre of sphere and the GPS antenna phase centre. The spheres were located close to the water line, and the distances from the boat were 10–30 m. In addition, the spheres were placed evenly throughout the test site and on both sides of the river.

## 3. Data Processing

**Figure 2.**
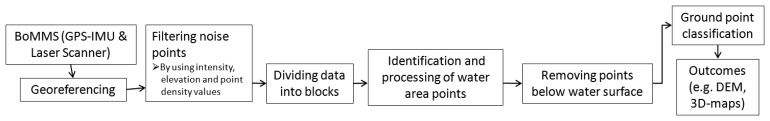
The processing chain for BoMMS point cloud data.

Noise point filtering, identification and processing of water area points and ground point classification were undertaken during data processing. The data processing chain for the terrain point classification is depicted in Figure 2. We converted profile laser data into georeferenced point clouds by assigning each of the mobile laser points the appropriate time stamp and coupling it with the trajectory information from the GPS-IMU system. The BoMMS uses the bi-trigger synchronization method, which delivers scanner triggers to the receiver log [44]. During the measurements, a time stamp was recorded for every laser scanning profile and further measurement time was interpolated for every scanning point. Waypoint Inertial Explorer software was used to compute the laser scanner trajectory [32].

After data acquisition, we employed three methods for filtering the non-object points of the georeferenced data (707 million points) from the 4.5 km trajectory. These methods were carried out individually on the 103 files collected.

### 3.1. Automatic Filtering Steps

**Figure 3.**
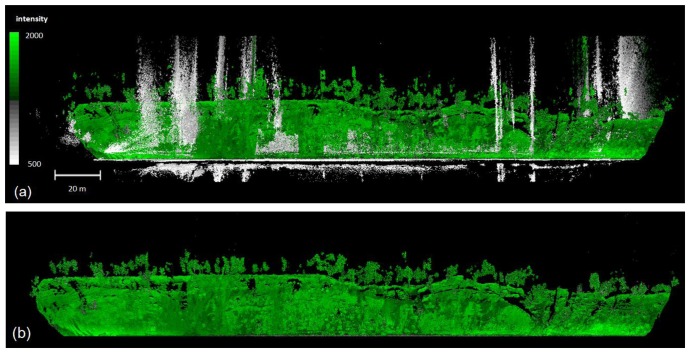
The intensity coloured point cloud and the front view of the river bank. (**a**) Data with noise points. (**b**) Data after noise point filtering and removal of the points below water surface.

Noise points are a typical feature in a point cloud because the system uses a phase difference scanner. In a river environment in particular, reflections from the water surface produce erroneous measurements below the ground and river bed. Our data also included some noise points above the ground surface (Figure 3). Typically, most noise points have a relatively low intensity value, in which case they can be filtered out by an intensity threshold. The appropriate threshold was determined from test sample so that it does not remove real observations from the targets and then we performed this filtering step for the entire dataset. The applied intensity threshold was 500 (scale 0–2,044). Some of the erroneous measurements were also filtered using absolute elevation by defining the lowest/highest object, *i.e.*, from benthic layer and tree canopy, and deleting the points below/above this defined elevation. In addition, the removal by range from the scanner is useful when the point cloud far from the scanner is not coherent due to obstacles. After intensity and absolute elevation based filtering, there were still noise points with low density. Hence, we removed the remaining erroneous measurements by computing the number of points within a certain radius in the air and removing the points if the density was less than the threshold. In our study, the system-specific threshold applied was 10 pts within a 50 cm radius sphere.

After the filtering steps, the data was divided into blocks (around 5–15 million points per block) for verifying the quality, cutting the points below water surface manually or repeating the point density based filtering method with different parameters and classifying the terrain points. The blocks were organized according to the trajectory. The filtered points contained 150 million points. Dividing the data into smaller block sizes makes the processing of larger projects more manageable.

### 3.2. Identification and Processing of Water Area Points

One part of data processing was the classification of water area points. These points can either be returns from the benthic layer or water column, or they can be noise or multipath reflections. By identifying MLS data characteristics related to water area, we can improve the quality of processed point clouds and separate water from land.

#### 3.2.1. Multipath Reflections

**Figure 4.**
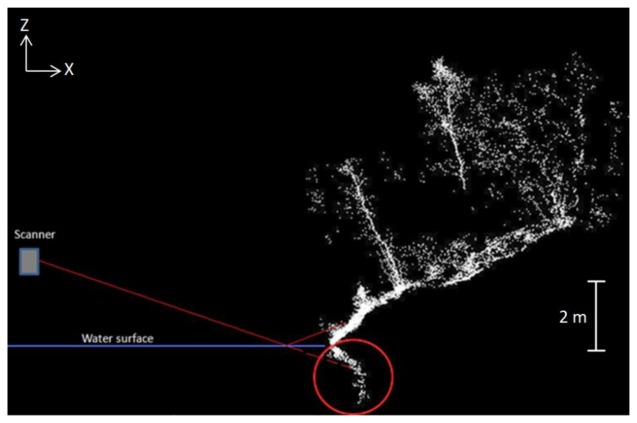
The point cloud profile demonstrates multipath reflections underneath the water surface.

Our measurements revealed that the onshore points can have mirrored points if the angle between water surface and laser beam is lower than the steepness of the ground (Figure 4). The multipath reflections produce extra points, which can be identified and removed locally by defining the height of the water surface. The effect appears in sloped terrain or because of the presence of vegetation near the shoreline. In this study, these points were removed manually. Otherwise, these points can also be removed automatically if the water surface can be estimated throughout the study area.

#### 3.2.2. Detection of Bathymetry Using BoMMS

**Figure 5.**
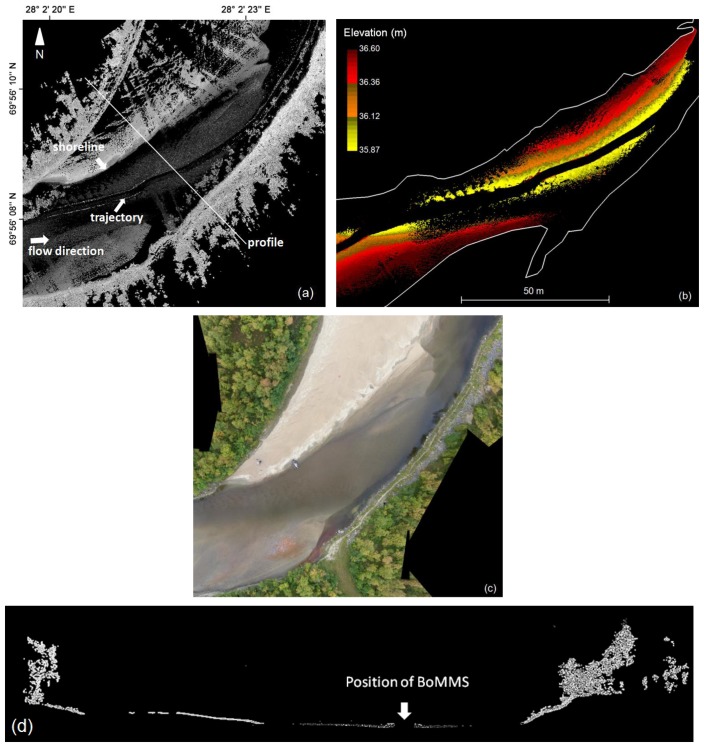
(**a**) Intensity image of the point cloud. The trajectory of the BoMMS is clearly visible as the occluded area in the middle of the river. The intensity value clearly changes at the border of the land and water areas; (**b**) The elevation coloured bathymetry points from the same area and digitized shoreline; (**c**) UAV mosaic; (**d**) Cross-sectional profile of the channel and point bar (location of the profile is marked on (**a**)).

**Figure 6.**
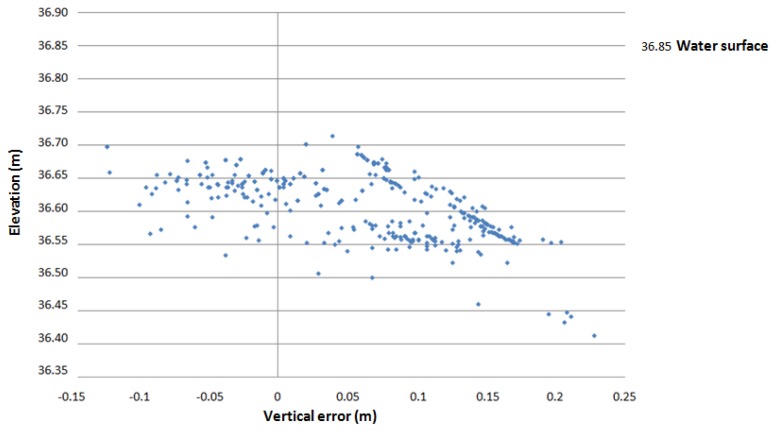
Vertical errors of MLS bathymetry points. ADCP measurements were used to provide reference data. The elevation of the water surface was detected with ADCP to be around 36.85 m. There are no ADCP points for the first 18 cm of depth.

The use of red and infrared laser beam for collecting bathymetric data is limited because at these wavelength lasers do not penetrate the water column very well. However, in this study, we intend to show that it is possible to acquire bathymetry data (Figures 5 and 6) at a wavelength of 785 nm (Faro Photon 120 scanner) in shallow (0–1 m) and clear water when the scanner is mounted on boat and near the water surface. The incidence angle at water surface varied approximately between 25° and 80° concerning bathymetry data. The smaller than 25° angles were occluded by boat.

We used 337 independent bathymetry elevation points surveyed with an Acoustic Doppler Current Profiler (ADCP) to provide reference data for the BoMMS. The comparison area consisted of sandy sediments. The grain sizes are analyzed by Kasvi *et al.* [40], varying mostly between 0.1 and 2 mm. The sensor was mounted on a remotely controlled miniboat. The measurement locations were measured with VRS-GPS (1 Hz) and the xy-coordinates were merged with the ADCP depth data in post-processing. The GPS and ADCP measurements had a maximum of 0.5 s time difference, which could cause a horizontal error of up to 10 cm. The ADCP measures depths greater than 0.18 m and its vertical beam sonar is rated to have a maximum error of 2.5% of depth. In addition, the MLS points were uncorrected in relation to the refraction of light at the water surface and the speed of light in water. The elevation difference (MLS-ADCP) is plotted against the elevation derived from ADCP measurements (Figure 6). Vertical errors ranged from 0.228 to −0.123 m, with a mean of 0.061 m and a standard deviation of 0.076 m. If the resulting bathymetry point cloud is to be used for DEM production, the data will require correction for refraction before registration [45]. We classified these points manually as they were clearly distinct from the dry areas or by determining the shoreline if the point cloud continued under the water surface (see Figure 5b).

#### 3.2.3. Shoreline Detection

**Figure 7.**
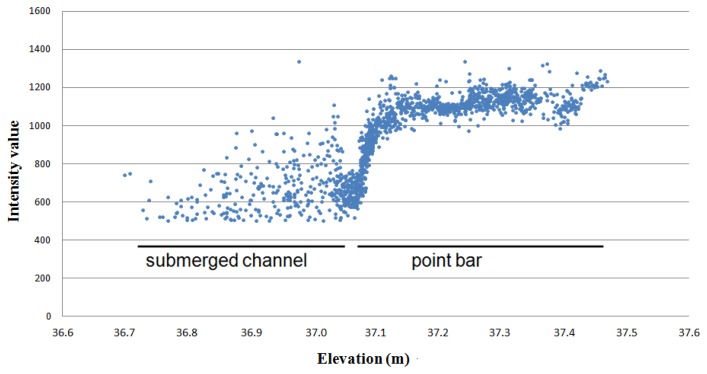
The profile of MLS points on the water–ground boundary indicates that intensity distribution is useful for identifying the shoreline from MLS data. For comparison, the ADCP retrieved water level was approximately 37.05 m.

The shoreline needed to be determined in the relatively flat areas where the point cloud continued under the water surface. For these cases, the shoreline was determined manually by identifying the intensity change at the water-ground boundary (Figures 5a,b and 7). The typical intensity value used to delineate the shoreline, ranged from 600 to 800 (scale 0–2,044). The determination was partly disrupted by the vegetation growing near the shoreline and above the water surface. In that case, the determination of the shoreline was easier if the potential shoreline points were initially classified by using the elevation value, e.g., selecting the points ±0.5 m from the estimated water surface.

### 3.3. Terrain Classification

Ground point classification was determined using the method described in Axelsson [13]. The method has achieved competitive results in ground filtering tests [20,21]. Meng *et al.* [21] reported that the method outperformed in comparison to nine other ground filters on sites with rough terrain or discontinuous surfaces. The method was originally developed for DEM generation from airborne laser data, but in this paper we assess the performance of the method for BoMMS data. The algorithm classifies terrain points by iteratively building a triangulated surface model. The method starts by selecting some seed points that are high probability ground points. These points are selected within a user-defined grid (parameter 1). The algorithm assumes that the grid area will have at least one hit on the ground and that the lowest point is a ground hit. Hence, the algorithm requires a careful filtering of erroneous points below the ground surface before execution. Seed points are selected to form an initial model. The routine then starts to densify the model by iteratively adding new laser points to it. In each iteration, a point is added to the model if the point meets certain criteria in relation to the triangle that contains it. The criteria are that the angle (parameter 2) a point makes to the triangle must be below a certain threshold and the point must be within a maximum distance (parameter 3) of the nearest triangle node. At the end of each iteration, the TIN and the data-derived thresholds are recomputed. The iterative process ends when there are no more points below the threshold.

## 4. Evaluation of Data Quality

### 4.1. Accuracy Assessment of the Ground Point Classification

**Figure 8.**
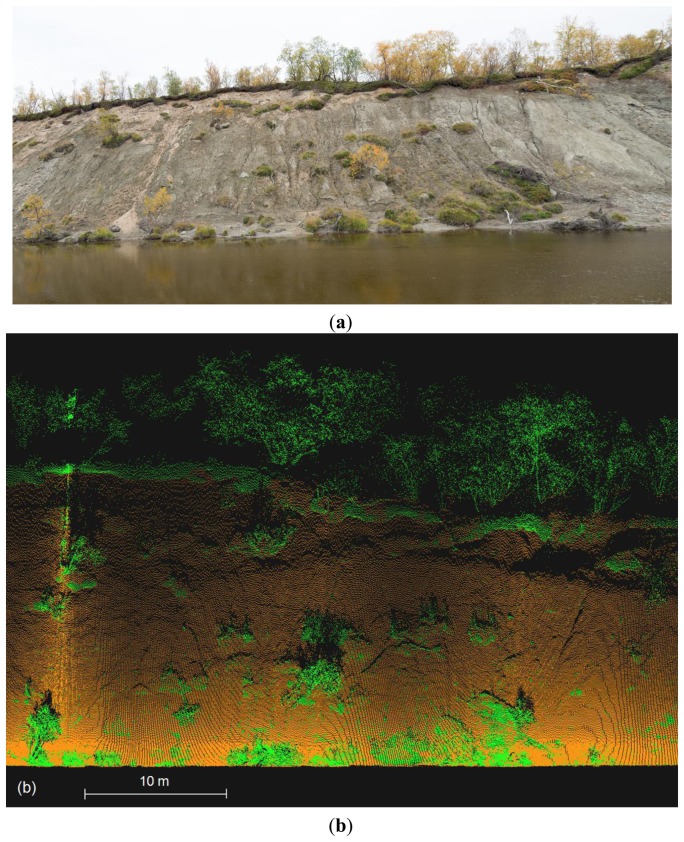
(**a**) The test site for the classification accuracy assessment was an erosion-sensitive and sparsely vegetated river bank with the height of 15–20 m; (**b**) Front view of the classified points in case 3. Ground points in orange and vegetation in green.

One of the most useful properties of the BoMMS system in the river environment is the mapping of topography changes on erosion-sensitive point bars and banks. Therefore, we evaluated the ground point classification accuracy on a steep (around 30–40 degree) and sparsely vegetated bank (Figure 8). The size of the test site was around 25 × 50 m. The data set totals of 323,966 points, consisting of 282,373 manually classified ground points and 41,593 vegetation points. Our analyses showed that the grid size parameter (see Section 3.3) had only a minor effect on the result of the ground classification when the parameter varied from 2 to 30 m. The biggest impact on the classification result was due to the iteration angle parameter. The results of automatic classification were compared with the manually classified dataset in order to determine which parameters perform best (Table 2). In the example, the grid size parameter is set to be constant (10 m), the iteration angle parameter varies from 10 to 40 degrees and the iteration distance parameter is 0.2 or 0.5 m for each angle.

We use the balanced accuracy (*ba*) measure [46,47] to assess the performance of the classification as:

(3) $$ba=\frac{1}{2}(a_{g}+a_{v})\;\text{with}$$

(4) $$a_{g}=\frac{tg}{tg+fv}$$

(5) $$a_{v}=\frac{tv}{tv+fg}$$

where *a_g_* and *a_v_* are class accuracies with *tg*, *tv*, *fg* and *fv* the number of points truly/falsely classified into the ground/vegetation classes. The results indicate that the ground point class accuracy has a tendency to increase when the value of the iteration angle increases. Simultaneously, the vegetation point class accuracy decreases. The best balanced accuracies of around 90% were achieved with the iteration distance of 0.2 m.

### 4.2. Data Features and Accuracy Assessment of z- and xy-Coordinates

**Figure 9.**
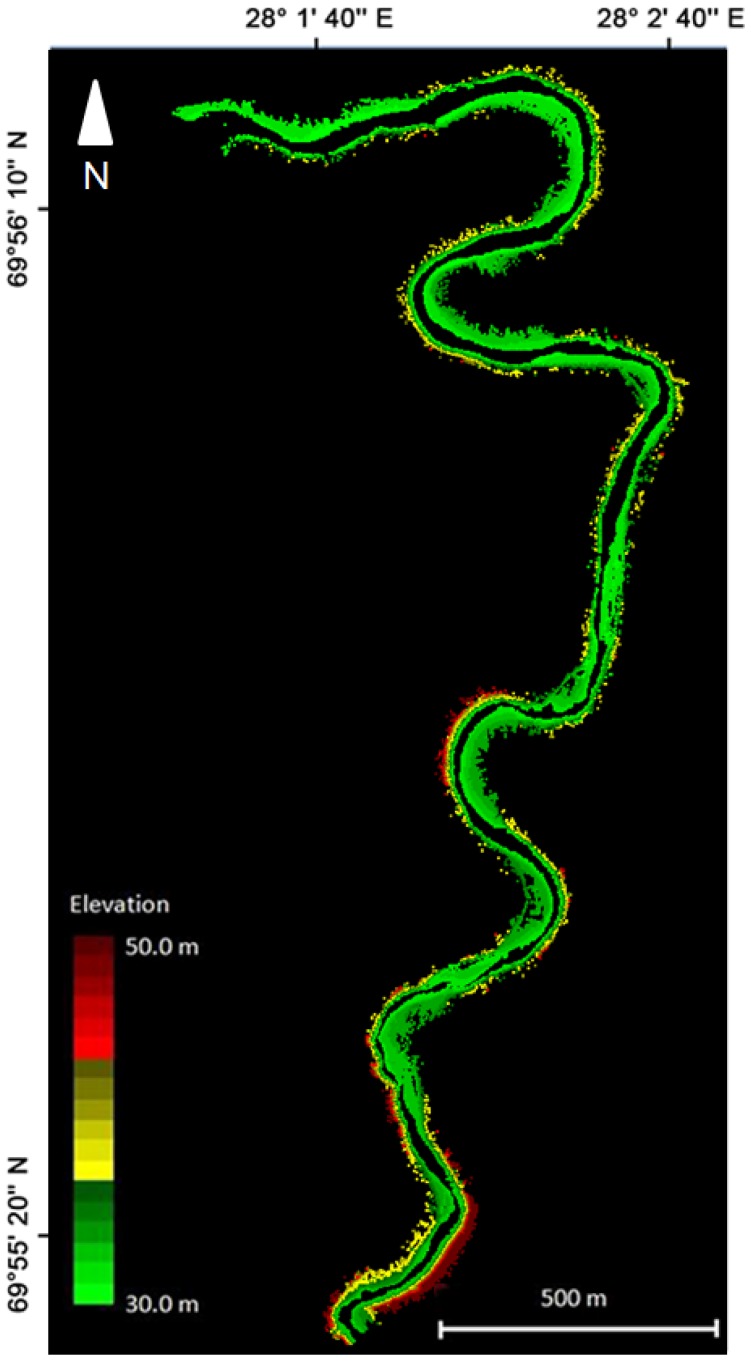
The river bank ground points of the BoMMS from a 4.5 km trajectory.

**Figure 10.**
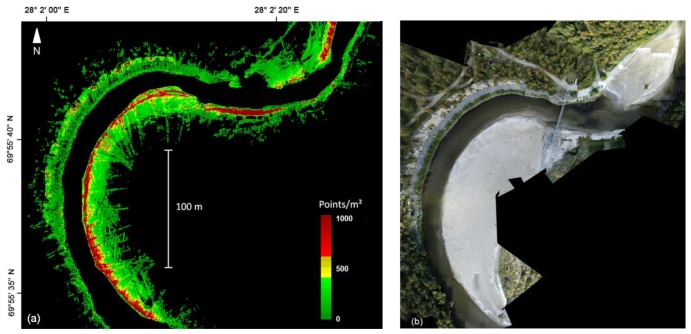
(**a**) The river bank and point bar point density map of classified ground points from the BoMMS data; (**b**) UAV-image mosaic of the same area.

The terrain points generated after the BoMMS data processing chain (see Figure 2) contained 10.8 million points for the 0.15 km^2^ study site (Figure 9) or 1.4% of the raw point cloud. The average density of the final ground point cloud was 72 pts/m^2^. However, the point density is irregular and strongly dependent on the scanning range (Figure 10). In this study, the ground point density varied mostly between 10 and 1,000 pts/m^2^. The presented point spacing Equations (1) and (2) can be used for calculating the point density at a certain range.

We evaluated the elevation and horizontal quality of the point cloud data against 16 spherical targets. The placement and measurements of the spheres are described in Section 2.3. Table 3 summarizes the accuracies of the z- and xy- values for the directly georeferenced BoMMS data. Minimum, maximum, mean and Root Mean Squared Error (RMSE) were computed for elevation and horizontal assessment. The z errors are real errors, including direction relative to the target points, whereas the xy errors are absolute values of distance. A comparison was performed by fitting a sphere to the laser points and computing the difference between the fitted sphere center and a reference sphere center measured by RTK-GPS.

## 5. Discussion

The findings of this study present new information regarding the data processing, characteristics and quality of BoMMS data in river environments. The presented processing chain was used for generating ground and vegetation points. Furthermore, we studied BoMMS's capability to measure bathymetry data.

We used three filtering method for removing the noise points from BoMMS data. These methods exploit intensity, absolute elevation, and point density within a certain radius for filtering erroneous measurements. The filtering methods can be performed automatically if the appropriate thresholds were determined from the test sample (see Section 3.1). It is important to pay attention to the reflection from the water surface when laser scanning from a boat platform. Effects such as multipath reflection will have a great impact on the quality of surface models, if it has not been taken into account during the data processing. We identified this effect near the shoreline (Section 3.2.1) and removed these points manually using profiles that are perpendicular to the direction of the system trajectory (Figure 4).

The identification and processing of the points below the water surface was one step in the processing chain. The MLS system used in this study showed a limited capability to estimate bathymetry information in shallow (0–1 m) and clear water. The bathymetry points were evaluated by using ADCP measurements as a reference. The elevation differences had a mean of 0.061 m and a standard deviation of 0.076 m. Milan *et al.* [48] reported that the resulting error in uncorrected bathymetry points can be depth dependent. The systematic error arises from refraction at the air-water interface [45]. The returns should be validated to ensure that they are coming from the benthic layer and not from the water column or the water surface. In addition, suitable incidence angles must be studied in more detail. The method has limitations, but the results indicate that it may be possible to use laser range measurement for collecting bathymetry data. In particular, the use of the green wavelength laser could improve the results because water absorbs green wavelengths at a slower rate than red wavelengths. In earlier studies related to laser scanning, the use of the green wavelength laser has been implemented in airborne bathymetry systems (e.g., [49]). Smith *et al.* [45] have studied the use of static through-water TLS (532 nm wavelength). In that study, a wide range of potential applications and limiting factors are discussed. In addition, we show that intensity values are useful for detecting the shoreline in MLS data (Section 3.2.3). The use of intensity values to differentiate wet and dry areas is also demonstrated by Milan and Heritage [50].

The last step of our data processing chain was a ground point classification. We used an iterative TIN-based method to classify ground points [13]. The results were compared to the manually classified data and we achieved a 90% balanced accuracy rate for separating ground and vegetation points. The most difficult landscapes to classify were rocky areas, small channels or cracks on the surface and areas, where the slope of the ground is near vertical. The selection of classification parameters also played a significant role in the performance of the classification. The classification and filtering algorithms developed for ALS data could be used for MLS if higher point density and different scanning geometry were taken into account. However, there is still a need to develop methods which are more suitable for MLS data.

In this work, the river environment was measured by one trajectory and our approach was to use spherical reference targets for controlling MLS data quality. The presented results of the z- and xy-coordinates indicate very small errors. The RMSE of the z- and xy-accuracies of the BoMMS point cloud were 1.0 and 2.3 cm, respectively, in comparison to the reference sphere targets. However, by using for example a TLS point cloud as reference [39], the systematic tendencies of elevation error can be identified spatially through error surfaces. Furthermore, overlapping scanning lines or point clouds could be used for evaluating measurement repeatability (*i.e.*, [32]). Our map of the spatial variations of the ground point density (Figure 7) indicates that the density is strongly dependent on the scanning range. Other factors affecting the point density are the measurement speed, scanning frequency and point measurement rate. The results show that after classification the ground point density varied between 10 and 1,000 pts/m^2^.

The MLS approach allowed an effective survey angle for sloped river banks, which is difficult to achieve with airborne or terrestrial scanning [38]. Mapping range and data coverage of BoMMS are dependent on line of sight visibility. In the present study, the maximum mapping range was around 50 m. If the data coverage needs to be enlarged, a low-altitude airborne system and a backpack MLS [32] would be a viable option. In recent years, UAV-based remote sensing has also provided promising results for environmental mapping. In riverine environments, the necessity to fly within line-of-sight might become a limitation for these systems. BoMMS can provide a more effective way to acquire data. UAV-based photography might also have difficulties in finding sufficient features for matching in areas of complex vegetation or homogeneous texture [51].

## 6. Conclusions

We developed a new BoMMS data processing chain that was shown to be capable of producing terrain and vegetation points from river environment by using automatic methods if the data characteristics such as intensity, point density and multipath reflections are identified. We obtained fairly good accuracy (90% balanced accuracy rate) for BoMMS ground point classification compared to manually classified data. Our evaluation of data quality also demonstrated the spatial variations of the ground point density. The accuracy assessment of BoMMS data indicated absolute accuracy level of some centimetres (horizontal RMSE 2.3 cm and elevation 1.0 cm). We demonstrated the use of intensity data for delineating the shoreline. Furthermore, the study indicates a possibility to collect bathymetry information in shallow (0–1 m) and clear water when the scanner is mounted on a boat and near the water surface. In further studies, the generated data is used to map changes in topography and vegetation structure [52,53]. We presume that future research dealing with LS data will increasingly turn to multi-temporal data investigation, updating different databases and change analysis applications. Hence, the development of effective data management, quality assessment, and of registration and monitoring methods for larger data sets will need to be considered as well.

## Figures and Tables

**Table 1.** System parameters of BoMMS in 2010.

**Scanner**	**FARO Photon 120**
Point measurement rate/Pulse repetition frequency	244 kHz
Scanning frequency	49 Hz
Point spacing of adjacent points in profile at 3/25/50 m	3.8 mm/3.2 cm/6.3 cm
Along-track point spacing for speeds of 1–2 m/s	2.0–4.1 cm

**Table 2.** Ground classification results of BoMMS data. The reference points include 282,373 manually classified ground points and 41,593 vegetation points.

**Case**	**Grid Size (m)**	**Iteration Angle (deg)**	**Iteration Distance (m)**	**Truly Classified Ground Points**	**Truly Classified Vegetation Points**	***a****_g_***(%)** (Equation (4))	***a****_v_***(%)** (Equation (5))	**Balanced Accuracy (%)** (Equation (3))
1	10	20	0.2	241,310	39,178	85.5	94.2	89.8
2	10	20	0.5	242,545	38,138	85.9	91.7	88.8
3	10	30	0.2	251,657	37,837	89.1	91.0	90.1
4	10	30	0.5	252,936	35,210	89.6	84.7	87.1
5	10	35	0.2	257,451	36,800	91.2	88.5	89.8
6	10	35	0.5	260,488	32,643	92.3	78.5	85.4
7	10	40	0.2	263,660	35,486	93.4	85.3	89.4
8	10	40	0.5	266,877	29,591	94.5	71.1	82.8

**Table 3.** The z- and xy-accuracies of the BoMMS point cloud compared to 16 reference sphere targets.

**Parameter**	**Min (m)**	**Max (m)**	**Mean (m)**	**RMSE (m)**
z	−0.019	0.022	0.005	0.010
xy	0.008	0.049	0.021	0.023
